# Low‐dose aspirin and risk of gastric and oesophageal cancer: A population‐based study in the United Kingdom using The Health Improvement Network

**DOI:** 10.1002/ijc.33022

**Published:** 2020-05-07

**Authors:** Luis A. García Rodríguez, Montse Soriano‐Gabarró, Pareen Vora, Lucía Cea Soriano

**Affiliations:** ^1^ Spanish Centre for Pharmacoepidemiologic Research (CEIFE) Madrid Spain; ^2^ Department of Epidemiology Bayer AG Berlin Germany; ^3^ Department of Public Health and Maternal and Child Health, Faculty of Medicine Complutense University of Madrid Madrid Spain

**Keywords:** cohort, gastric cancer, low‐dose aspirin, nested case‐control, oesophageal cancer

## Abstract

There is increasing interest regarding potential protective effects of low‐dose aspirin against various gastrointestinal cancers. We aimed to quantify the association between use of low‐dose aspirin and risk of gastric/oesophageal cancer using a population‐based primary care database in the UK. Between January 2005 and December 2015, we identified a cohort of 223 640 new users of low‐dose aspirin (75‐300 mg/day) and a matched cohort of nonusers at the start of follow‐up from The Health Improvement Network. Cohorts were followed to identify incident cases of gastric/oesophageal cancer. Nested case‐control analyses were conducted and adjusted odds ratios (ORs) with 95% confidence intervals (CIs) were calculated for current vs nonuse of low‐dose aspirin using logistic regression. Current use was defined as when low‐dose aspirin lasted 0 to 90 days before the index date (event date for cases, random date for controls) and previous duration was ≥1 year. We identified 727 incident cases of gastric cancer and 1394 incident cases of oesophageal cancer. ORs (95% CIs) were 0.46 (0.38‐0.57) for gastric cancer and 0.59 (0.51‐0.69) for oesophageal cancer. The effect remained consistent with no clear change seen between previous duration of low‐dose aspirin use of 1‐3, 3‐5 or >5 years. The reduced risks was seen with 75 mg/day, and effects were consistent in lag‐time analyses. In conclusion, our results indicate that use of low‐dose aspirin is associated with a 54% reduced risk of gastric cancer and a 41% reduced risk of oesophageal cancer as supported by mechanistic data.

## Abstract 2

**What's new?**

Low‐dose aspirin may help protect against the development of gastric and oesophageal cancers. This population‐based study using data from primary care electronic health records in the United Kingdom lends further support to that idea. Compared with no use of low‐dose aspirin, daily use of low‐dose aspirin (75–300 mg) for at least one year was associated with a 54 percent reduction in gastric cancer risk and a 41 percent reduction in oesophageal cancer risk. No clear difference in effect was observed between low‐dose aspirin use for one to three years versus more than three years.

**Abbreviations**
BMI: body mass index
CI: confidence intervals
COX: cyclooxygenase
CRC: colorectal cancer
GP: general practitioner
HES: Hospital Episode Statistics
OR: odds ratios
OTC: over‐the‐counter
RCT: randomised controlled trials
THIN: The Health Improvement Network
UK: United Kingdom

## 1 INTRODUCTION

In addition to the benefits of daily low‐dose aspirin for the prevention of cardiovascular disease (CVD), there is strong evidence from meta‐analysis of CVD randomised controlled trials (RCTs) and from observational studies that daily low‐dose aspirin reduces the risk of colorectal cancer (CRC) by about 30% to 40%.^1^, ^2^, ^3^, ^4^, ^5^, ^6^, ^7^ Several lines of evidence indicate that the central mechanism for low‐dose aspirin's protective effect against CRC—the permanent inhibition of cyclooxygenase (COX)‐I enzyme in platelets, leading to suppression of thromboxane A_2_ synthesis and reduced platelet activation—is the same through which its cardiovascular benefits are mediated.^8^, ^9^

Although most evidence for low‐dose aspirin effects in the prevention of cancer is for CRC, there is increasing interest in the chemoprotective effects of low‐dose aspirin against other gastrointestinal cancers, in particular gastric and oesophageal cancer. These effects may be mediated through a similar mechanism of action on the gastrointestinal mucosa as with CRC.^8^ Survival rates of these two cancers—the fifth and seventh most commonly diagnosed cancers worldwide, respectively,^10^ are poor^11^, ^12^, ^13^, ^14^ with diagnosis commonly at advanced stage at presentation. In the United Kingdom (UK), over half of patients have stage III or IV disease at diagnosis^15^, ^16^ and only around half are still alive 1 year postdiagnosis.^12^, ^13^ Further chemoprotective benefits of low‐dose aspirin, in addition to the established benefits in CVD and CRC prophylaxis, could therefore potentially further improve the overall balance of benefits and bleeding risks for low‐dose aspirin. In an analysis of three aspirin CVD trials with 20‐year posttrial follow‐up,^17^, ^18^, ^19^ Rothwell et al^20^ found that low‐dose aspirin use at baseline was associated with a 60% reduction in death from oesophageal cancer and a 30% reduction in death from gastric cancer.

The majority of observational studies similarly support substantial reductions in risk of mortality^3^ and incidence^3^, ^21^, ^22^, ^23^, ^24^, ^25^ of gastric/oesophageal cancer with low‐dose aspirin use, yet confidence in the existing data would be strengthened by similar findings from further population‐based studies with a robust study design to minimise bias and confounding. Furthermore, questions remain regarding the effects of low‐dose aspirin duration and dose on gastric/oesophageal cancer risk.

## 2 MATERIALS AND METHODS

### 2.1 Study design

Using primary care data from the UK, we conducted a population‐based cohort study involving the follow‐up of a cohort of new‐users of low‐dose aspirin (75‐300 mg/day) and a matched cohort of nonusers of low‐dose aspirin at the start of follow‐up to identify incident cases of gastric and oesophageal cancer. The matching served to minimise bias caused by differences between users and nonusers of low‐dose aspirin at the time that treatment was initiated. As exposure to low‐dose aspirin can change during a long follow‐up period, we subsequently performed a nested case‐control analysis to more accurately evaluate the effect of low‐dose aspirin exposure over time. Our study design has been implemented in previous studies evaluating the associations between low‐dose aspirin use and clinical outcomes.^4^, ^26^, ^27^

### 2.2 Data sources

We used The Heath Improvement Network (THIN) primary care database, which is validated and representative of the UK demographic.^28^, ^29^ The database contains the longitudinal electronic health records (EHRs) of approximately 6% of the UK population, with approximately 3 million individuals currently alive and registered with a participating general practice. The database captures information recorded by general practitioners (GPs) and other practice staff as part of routine patient care. Patient data are entered using the Read code system—the comprehensive coded thesaurus used by clinicians in the UK.^30^ Information from secondary care received via email or letter is also recorded. The Health Improvement Network is a valid data source for studying low‐dose aspirin use in the UK because all prescriptions issued by GPs are automatically recorded in the patient's EHR, and while low‐dose aspirin is available over‐the‐counter (OTC) in the UK, we have previously shown that misclassification of low‐dose aspirin in THIN due to unrecorded use of OTC low‐dose aspirin is minimal.^31^ Acquisition of THIN data was provided to the Centro Español de Investigación Farmacoepidemiológica (CEIFE) by IQVIA under the UK's National Health Service South‐East multicenter research committee approval in 2003. The study protocol was approved by an Independent Scientific Research Committee for THIN (reference 17THIN059_A1). No individual patient consent was required because the study used de‐identified data provided by patients as a part of their routine primary care.

### 2.3 Identification of the study cohorts

A flowchart depicting the study design is shown in Figure S1. We identified all individuals aged 40 to 89 years in THIN between January 1, 2005 and December 31, 2015 (study entry period) with at least 2 years’ registration with their GP and at least 1 year of recorded prescription history. The date an individual met these eligibility criteria was the study entry date. Individuals with a prescription for low‐dose aspirin (n = 417 256) or with a record of cancer before this date (n = 223 274) were excluded. We subsequently identified patients (n = 223 640) with a first prescription for low‐dose aspirin (75‐300 mg/day; start date) and matched each 1:1 to a nonuser of low‐dose aspirin on their start date by age, sex, time since study entry, and number of GP visits in the previous year. This process resulted in two cohorts: new users of low‐dose aspirin and nonusers of low‐dose aspirin at the start of follow‐up. The start date for a member of the latter cohort was the start date of their matched partner in the low‐dose aspirin cohort. All members of both cohorts were assigned to either the primary or secondary CVD prevention population based on whether they had a Read code for CVD before the start date, as described previously.^32^

### 2.4 Follow‐up and outcome identification

We performed two independent follow‐ups, one to identify first time cases of gastric cancer and the other to identify first time cases of oesophageal cancer. Individuals were followed until the earliest of the following endpoints: a recorded diagnosis of gastric/oesophageal cancer (based on codes specific for these cancers; see Tables S1 and S2 for Read codes), a recorded diagnosis of another cancer, death or the end of follow‐up (31 December 2017). A patient was designated as confirmed/unconfirmed case following a stepwise process (Supporting Information Methods) involving manual review of patient records. A fatal case was defined as death from any cause within 1 year after the index date. The index date for all confirmed cases was the date of the recorded diagnosis of gastric/oesophageal cancer.

### 2.5 Selection of controls

Controls were selected from both the low‐dose aspirin and matched nonuser study cohorts using incidence density sampling and were frequency matched to cases by age, sex and calendar year (n = 5000 each for cases of both cancers). The index date for controls was a random date during the individual's observation period.

### 2.6 Low‐dose aspirin exposure

The length of supply in days of a low‐dose aspirin prescription was calculated as the number of tablets prescribed divided by the prescribed daily posology. Although we did not have information on adherence to treatment, we assumed that patients took their medication daily as prescribed. Current use of low‐dose aspirin among cases and controls was defined as when supply of the most recent prescription before the index date lasted until/over the index date or ended 0 to 30 days before the index date. In our analysis of “current use”, we required current users to have at least 1 year of low‐dose aspirin use because those with short durations (ie, those who recently initiated low‐dose aspirin) of use may be prone to confounding by contraindication; early signs of gastric cancer manifesting as gastrointestinal symptoms that may cause the GP to withhold prescribing low‐dose aspirin. Remaining current users were those with a treatment duration of less than 1 year. Other categories of low‐dose aspirin exposure were *recent use*, when supply of the most recent prescription ended 31 to 365 days before the index date; *past use* was when supply of the most recent prescription ended ≥365 days before the index date, and *nonuse*, when there was no recorded use at any time before the index date. Duration of low‐dose aspirin was calculated by summing the duration of individual prescriptions before the index date. Low‐dose aspirin dose was computed based on the posology of the last prescription before the index date.

### 2.7 Covariables

Patient demographics and data on lifestyle factors, healthcare use, comorbidities and other medications were extracted from the database. Information on age, sex and lifestyle factors (body mass index [BMI in kg/m^2^], smoking status and alcohol consumption) were collected any time before the index date using the most recent value/status as appropriate. For BMI, we discounted values calculated using weight data recorded in the year before the index date to avoid using measurements affected by weight loss due to cancer. Comorbidities were identified any time before the index date, and healthcare use (number of GP visits in the year before the index date) were established in the year before the index date. A proxy variable for *Helicobacter pylori* infection was created based on a having at least a Read code for *H. pylori* gastritis, confirmation of the infection from a laboratory test or a code relating to eradication therapy. Use of medications other than low‐dose aspirin was identified in the year before the index date, with current use defined as per the definition for low‐dose aspirin irrespective of duration of treatment. Treatment for *H. pylori* infection (as a separate variable to the proxy variable for *H. pylori* described above) was identified any time before the index date.

### 2.8 Statistical analysis

For both gastric and oesophageal cancers, we performed nested case‐control analyses using cases and controls from both cohorts to estimate the association between use of low‐dose aspirin (as well as the demographics, lifestyle factors, comorbidities and co‐medication variables as described above) with the risk of gastric/oesophageal cancer. Odds ratios (ORs) with 95% confidence intervals (CIs) were calculated using unconditional logistic regression adjusted for confounders (smoking, number of GP visits in the year before the index date and low‐dose aspirin [in analyses of all other variables] in addition to the matching factors of age, sex and calendar year). We used a stepwise approach adding potential confounders to the unadjusted model, retaining those that changed the estimate by at least 10%. As controls were selected using incidence density sampling, the ORs were regarded as an unbiased estimate of the incidence rate ratio. Stratified analyses were performed by primary/secondary CVD prevention population, and further analyses were undertaken according to the duration and dose of low‐dose aspirin therapy. We also analysed concomitant use of low‐dose aspirin with clopidogrel, with nonsteroidal anti‐inflammatory drugs, and with proton pump inhibitors (PPIs), and undertook lag‐time analyses backdating the index date by 1 year to account for the cancer latency periods. STATA version 12.0 was used for all analyses.

## 3 RESULTS

A total of 826 individuals were identified with a first diagnosis of gastric cancer and 1523 individuals were identified with a first diagnosis of oesophageal cancer during a mean follow‐up of 5.47 years (both follow‐ups). After the multistep case confirmation process, there were 727 incident cases of gastric cancer and 1394 incident cases of oesophageal cancer (confirmation rates of 88.0% and 78.7%, respectively). Among cases of gastric cancer, 68.0% were among men, the median age was 74.0 years (inter‐quartile range [IQR] 67.0‐80.0), the mean age was 73.4 years (SD 9.6 years) and 58.3% were fatal. Among cases of oesophageal cancer, 73.7% were among men, the median age was 71.0 years (IQR 64.0‐78.0), the mean age was 70.9 years (SD 10.0 years) and 53.7% were fatal.

### 3.1 Baseline characteristics of cases and controls

Baseline characteristics of cases and controls are shown in Table 1 for gastric cancer and Table 2 for oesophageal cancer. A high level of primary care visits, smoking, anaemia, pernicious anaemia, peptic ulcer, use of acid suppressive medications and treatment for *H. pylori* infection were all associated with an increased risk of gastric cancer. A significantly reduced risk of gastric cancer was seen with current use of nonsteroidal anti‐inflammatory drugs (NSAIDs). Factors associated with an increased risk of oesophageal cancer included oesophageal ulcer (nearly nine‐fold increased risk), oesophageal varices, a high level of primary care visits, nonpernicious anaemia, smoking, high alcohol consumption (≥42 U/week), and use of acid‐suppressive agents. Frailty, previous lower gastrointestinal bleeding, ischaemic heart disease, hypertension, diabetes, hyperlipidaemia and use of NSAIDs were all associated with a reduced risk of oesophageal cancer.

**TABLE 1:** Odds ratios (95% CIs) for the association between patient characteristics and the risk of gastric cancer

	Cases (n = 727)		Controls (n = 5000)			
	n	%	n	%	OR (95% CI)^a^	OR (95% CI)^b^
Sex						
Male	494	68.0	3387	67.7	—	—
Female	233	32.0	1613	32.3	—	—
Age at index date (years)						
40‐49	14	1.9	93	1.9	—	—
50‐59	44	6.1	305	6.1	—	—
60‐69	154	21.2	1066	21.3	—	—
70‐79	303	41.7	2072	41.4	—	—
≥80	212	29.2	1464	29.3	—	—
GP visits						
0‐4	21	2.9	574	11.5	1.0 (reference)	1.0 (reference)
5‐9	87	12.0	1235	24.7	1.98 (1.22‐3.23)	1.98 (1.21‐3.23)
10‐19	292	40.2	1931	38.6	4.30 (2.73‐6.78)	4.21 (2.67‐6.64)
≥20	327	45.0	1260	25.2	7.52 (4.76‐11.86)	7.33 (4.64‐11.57)
BMI (kg/m^2^)						
15‐19 (underweight)	30	4.1	162	3.2	1.26 (0.83‐1.92)	0.99 (0.65‐1.53)
20‐24 (healthy weight)	201	27.6	1353	27.1	1.0 (reference)	1.0 (reference)
25‐29 (overweight)	274	37.7	1944	38.9	0.95 (0.78‐1.15)	0.93 (0.76‐1.14)
≥30 (obese)	166	22.8	1183	23.7	0.94 (0.75‐1.18)	0.82 (0.65‐1.03)
Missing	56	7.7	358	7.2	1.05 (0.76‐1.45)	1.32 (0.95‐1.84)
Smoking						
Nonsmoker	221	30.4	1955	39.1	1.0 (reference)	1.0 (reference)
Current	129	17.7	614	12.3	1.89 (1.49‐2.39)	1.78 (1.40‐2.28)
Former	376	51.7	2408	48.2	1.39 (1.16‐1.67)	1.24 (1.03‐1.49)
Missing	1	0.1	23	0.5	0.38 (0.05‐2.86)	0.48 (0.06‐3.64)
Alcohol consumption (units/week)						
None	133	18.3	940	18.8	1.0 (reference)	1.0 (reference)
1‐9	347	47.7	2245	44.9	1.08 (0.87‐1.34)	1.11 (0.89‐1.39)
10‐20	101	13.9	838	16.8	0.84 (0.63‐1.11)	0.87 (0.65‐1.17)
21‐41	29	4.0	284	5.7	0.70 (0.46‐1.08)	0.72 (0.46‐1.11)
≥42	18	2.5	112	2.2	1.11 (0.65‐1.90)	0.99 (0.57‐1.71)
Missing	99	13.6	581	11.6	1.19 (0.90‐1.58)	1.38 (1.03‐1.84)
Frailty						
Fit	249	34.3	2235	44.7	1.0 (reference)	1.0 (reference)
Mild frailty	293	40.3	1807	36.1	1.56 (1.30‐1.89)	0.98 (0.80‐1.20)
Moderate frailty	146	20.1	733	14.7	2.02 (1.59‐2.56)	0.99 (0.76‐1.29)
Severe frailty	39	5.4	225	4.5	1.82 (1.24‐2.67)	0.76 (0.50‐1.14)
Comorbidity						
Hypertension	670	54.6	2553	56.7	0.83 (0.71‐0.98)	0.69 (0.59‐0.82)
Hyperlipidemia	165	22.7	1210	24.2	0.92 (0.76‐1.11)	0.87 (0.72‐1.06)
Ischaemic stroke	37	5.1	318	6.4	0.79 (0.55‐1.12)	0.66 (0.46‐0.94)
IHD	131	18.0	916	18.3	0.98 (0.80‐1.20)	0.81 (0.66‐1.00)
Diabetes	127	17.5	871	17.4	1.00 (0.82‐1.23)	0.74 (0.60‐0.91)
Anaemia	95	13.1	126	2.5	5.95 (4.49‐7.89)	4.73 (3.54‐6.31)
Pernicious anaemia	16	1.1	23	0.5	4.19 (2.04‐8.62)	3.21 (1.54‐6.68)
Dyspepsia	274	37.7	1071	21.4	2.23 (1.89‐2.63)	1.89 (1.60‐2.24)
GORD	176	24.2	824	16.5	1.63 (1.35‐1.96)	1.35 (1.12‐1.64)
Hiatus hernia	50	6.9	338	6.8	1.02 (0.75‐1.39)	0.85 (0.62‐1.16)
Peptic ulcer^c^/UGIB/unspecified GIB	173	23.8	377	7.5	3.90 (3.18‐4.77)	3.25 (2.64‐4.00)
LGIB	55	7.6	387	7.7	0.98 (0.73‐1.31)	0.86 (0.64‐1.16)
Complicated peptic ulcer	67	9.2	145	2.9	3.44 (2.54‐4.66)	2.95 (2.16‐4.02)
Uncomplicated peptic ulcer	129	17.7	272	5.4	3.79 (3.02‐4.76)	3.19 (2.52‐4.03)
IBD	6	0.8	89	1.8	0.46 (0.20‐1.05)	0.37 (0.16‐0.86)
IBS	32	4.4	243	4.9	0.90 (0.62‐1.32)	0.74 (0.50‐1.09)
Proxy measure for *H. pylori infection*	56	7.7	130	2.6	3.14 (2.27‐4.34)	2.74 (1.96‐3.83)
Medications						
Clopidogrel	52	7.2	253	5.1	1.46 (1.07‐1.99)	1.15 (0.84‐1.58)
Oral anticoagulant	62	8.5	336	6.7	1.32 (0.99‐1.75)	0.88 (0.66‐1.18)
PPI	449	61.8	1261	25.2	5.92 (4.96‐7.08)	4.80 (3.99‐5.77)
H_2_RA	48	6.6	118	2.4	3.01 (2.13‐4.25)	2.69 (1.88‐3.84)
Antacid	105	14.4	261	5.2	3.11 (2.44‐3.97)	2.58 (2.01‐3.30)
NSAID	31	4.3	355	7.1	0.59 (0.40‐0.85)	0.50 (0.34‐0.74)
Treatment for *H. pylori*	29	4.0	87	1.7	2.35 (1.53‐3.60)	2.08 (1.34‐3.24)

*Note:* Comorbidities were identified any time before the index date except for anaemia, where we included recorded diagnoses in the year before the index date, and pernicious anaemia, where we included recorded diagnoses in the 5 years before the index date.

Abbreviations: BMI, body mass index; CI, confidence interval; COXIB, cyclooxygenase‐2 inhibitors, DVT, deep vein thrombosis; GIB, gastrointestinal bleeding; GORD, gastro‐oesophageal reflux disease; GP, general practitioner; H_2_RA, histamine_2_ receptor antagonist; IBD, irritable bowel disease; IBS, inflammatory bowel syndrome; IHD, ischaemic heart disease; LGIB, lower gastrointestinal disease; NSAID, nonsteroidal anti‐inflammatory drug; OR, odds ratio; PPI, proton pump inhibitor; TIA, transient ischaemic attack; tNSAID, traditional nonsteroidal anti‐inflammatory drug; UGIB, upper gastrointestinal bleeding.

a Adjusted by the matching factors: sex, age and calendar year.

b Adjusted by the matching factors: sex, age, and calendar year, and by smoking, number of GP visits in the year before the index date and current use of low‐dose aspirin.

c Includes both complicated and uncomplicated peptic ulcer.

**TABLE 2:** Odds ratio (95% CIs) for the association between patient characteristics and the risk of oesophageal cancer

	Cases (n = 1394)		Controls (n = 5000)			
	n	%	n	%	OR (95% CI)^a^	OR (95% CI)^b^
Sex						
Male	1027	73.7	3663	73.3	—	—
Female	367	26.3	1337	26.7	—	—
Age at index date (years)						
40‐49	17	1.2	63	1.3	—	—
50‐59	169	12.1	614	12.3	—	—
60‐69	428	30.7	1534	30.7	—	—
70‐79	492	35.3	1751	35.0	—	—
≥80	288	20.7	1038	20.8	—	—
GP visits						
0‐4	59	4.2	671	13.4	1.0 (reference)	1.0 (reference)
5‐9	242	17.4	1226	24.5	2.31 (1.71‐3.12)	2.31 (1.71‐3.13)
10‐19	581	41.7	1966	39.3	3.50 (2.64‐4.65)	3.58 (2.69‐4.78)
≥20	512	36.7	1137	22.7	5.48 (4.10‐7.32)	5.53 (4.13‐7.42)
BMI (kg/m^2^)						
15‐19 (underweight)	64	4.6	150	3.0	1.48 (1.08‐2.03)	1.34 (0.97‐1.86)
20‐24 (healthy weight)	369	26.5	1267	25.3	1.0 (reference)	1.0 (reference)
25‐29 (overweight)	503	36.1	1978	39.6	0.87 (0.74‐1.01)	0.87 (0.74‐1.02)
≥30 (obese)	353	25.3	1239	24.8	0.98 (0.82‐1.15)	0.91 (0.77‐1.09)
Missing	105	7.5	366	7.3	0.99 (0.77‐1.27)	1.25 (0.96‐1.62)
Smoking						
Nonsmoker	361	25.9	1887	37.7	1.0 (reference)	1.0 (reference)
Current	327	23.5	664	13.3	2.67 (2.24‐3.20)	2.71 (2.26‐3.25)
Former	700	50.2	2430	48.6	1.52 (1.32‐1.75)	1.40 (1.21‐1.62)
Missing	6	0.4	19	0.4	1.67 (0.66‐4.23)	2.42 (0.92‐6.36)
Alcohol consumption (units/week)						
None	197	14.1	786	15.7	1.0 (reference)	1.0 (reference)
1‐9	571	41.0	2211	44.2	1.04 (0.86‐1.24)	1.08 (0.89‐1.30)
10‐20	274	19.7	935	18.7	1.19 (0.96‐1.47)	1.21 (0.97‐1.50)
21‐41	109	7.8	313	6.3	1.42 (1.08‐1.87)	1.35 (1.02‐1.80)
≥42	67	4.8	150	3.0	1.83 (1.31‐2.55)	1.69 (1.20‐2.39)
Missing	176	12.6	605	12.1	1.17 (0.93‐1.47)	1.29 (1.02‐1.64)
Frailty						
Fit	624	44.8	2408	48.2	1.0 (reference)	1.0 (reference)
Mild frailty	511	36.7	1813	36.3	1.11 (0.97‐1.28)	0.74 (0.64‐0.86)
Moderate frailty	220	15.8	628	12.6	1.42 (1.17‐1.73)	0.78 (0.63‐0.96)
Severe frailty	39	2.8	151	3.0	1.07 (0.73‐1.55)	0.52 (0.35‐0.77)
Comorbidities						
Hypertension	729	52.3	2779	55.6	0.87 (0.77‐0.98)	0.75 (0.66‐0.85)
Hyperlipidemia	321	23.0	1241	24.8	0.90 (0.79‐1.04)	0.84 (0.72‐0.97)
Ischaemic stroke	86	6.2	286	5.7	1.08 (0.84‐1.39)	0.87 (0.68‐1.13)
IHD	213	15.3	923	18.5	0.79 (0.67‐0.93)	0.67 (0.57‐0.80)
Diabetes	256	18.4	939	18.8	0.97 (0.83‐1.13)	0.74 (0.63‐0.87)
Anaemia	61	4.4	130	2.6	1.72 (1.26‐2.35)	1.29 (0.94‐1.78)
Pernicious anaemia	16	1.1	23	0.5	2.53 (1.33‐4.80)	2.11 (1.09‐4.07)
Dyspepsia	462	33.1	1131	22.6	1.70 (1.49‐1.93)	1.50 (1.32‐1.72)
GORD	439	31.5	844	16.9	2.28 (1.99‐2.61)	2.11 (1.83‐2.43)
Hiatus hernia	157	11.3	292	5.8	2.05 (1.67‐2.52)	1.79 (1.45‐2.21)
Oesophageal ulcer	30	2.2	12	0.2	9.16 (4.68‐17.95)	8.79 (4.37‐17.68)
Oesophageal varices	6	0.4	5	0.1	4.30 (1.31‐14.12)	3.12 (0.93‐10.42)
Peptic ulcer/UGIB/unspecified GIB	125	9.0	350	7.0	1.31 (1.06‐1.62)	1.13 (0.91‐1.40)
Previous LGIB	82	5.9	397	7.9	0.72 (0.57‐0.92)	0.65 (0.50‐0.83)
Complicated peptic ulcer	63	4.5	141	2.8	1.63 (1.20‐2.21)	1.37 (1.00‐1.87)
Uncomplicated peptic ulcer	75	5.4	251	5.0	1.07 (0.82‐1.40)	0.94 (0.72‐1.23)
Proxy measure of *H. pylori*	57	4.1	146	2.9	1.42 (1.04‐1.94)	1.17 (0.85‐1.61)
Medications						
Clopidogrel	72	5.2	264	5.3	0.98 (0.75‐1.28)	0.79 (0.60‐1.03)
Oral anticoagulant	96	6.9	333	6.7	1.04 (0.82‐1.32)	0.73 (0.57‐0.94)
PPI	894	64.1	1201	24.0	6.96 (6.06‐7.99)	6.16 (5.33‐7.11)
H_2_RA	88	6.3	126	2.5	2.64 (2.00‐3.49)	2.34 (1.75‐3.11)
Antacid	269	19.3	237	4.7	4.90 (4.06‐5.91)	4.28 (3.53‐5.20)
NSAID	79	5.7	329	6.6	0.85 (0.66‐1.09)	0.77 (0.60‐1.00)
Treatment for *H. pylori*	23	1.7	85	1.7	0.97 (0.61‐1.54)	0.81 (0.50‐1.30)

*Note:* Comorbidities were identified any time before the index date except for anaemia, where we included recorded diagnoses in the year before the index date, and pernicious anaemia, where we included recorded diagnoses in the 5 years before the index date.

Abbreviations: BMI, body mass index; CI, confidence interval; COXIB, cyclooxygenase‐2 inhibitors, DVT, deep vein thrombosis; GIB, gastrointestinal bleeding; GORD, gastro‐oesophageal reflux disease; GP, general practitioner; H_2_RA, histamine_2_ receptor antagonist; IBD, irritable bowel disease; IBS, inflammatory bowel syndrome; IHD, ischaemic heart disease; LGIB, lower gastrointestinal disease; NSAID, nonsteroidal anti‐inflammatory drug; OR, odds ratio; PPI, proton pump inhibitor; TIA, transient ischaemic attack; tNSAID, traditional nonsteroidal anti‐inflammatory drug; UGIB, upper gastrointestinal bleeding.

a Adjusted by the matching factors: sex, age and calendar year.

b Adjusted by the matching factors: sex, age, and calendar year, and by smoking, number of GP visits in the year before the index date and current use of low‐dose aspirin.

### 3.2 Low‐dose aspirin use and risk of gastric cancer

Associations between low‐dose aspirin and risk of gastric cancer are shown in Table 3. Compared to nonuse of low‐dose aspirin, current use of low‐dose aspirin was associated with a 54% reduced risk of gastric cancer (OR 0.46, 95% CI: 0.38‐0.57), with estimates similar when stratified by primary/secondary CVD prevention. The reduced risk remained consistent with no clear change with increasing durations of use, and was evident at the lowest dose of aspirin evaluated (75 mg/day), which was the dose used by the vast majority of cases and controls. Lag‐time analyses showed minimal changes in the effects seen (Table S3). The reduced risk was also seen when low‐dose aspirin was used concomitantly with clopidogrel (OR 0.62, 95% CI: 0.27‐1.42) or NSAIDs (OR 0.27, 95% CI: 0.12‐0.58) vs nonuse of both drugs in each analysis. Concomitant use of low‐dose aspirin and a PPI, however, was associated with an increased risk of gastric cancer (OR 1.94, 95% CI: 1.45‐2.60).

**TABLE 3:** Odds ratios (95% CIs) for the association between low‐dose aspirin use and the risk of gastric cancer

	Cases (n = 727)		Controls (n = 5000)			
Low‐dose aspirin	%	%	n	%	OR (95% CI)^a^	OR (95% CI)^b^
Recency of use						
Nonuse	442	60.8	2404	48.1	1.0 (reference)	1.0 (reference)
Current use	146	20.1	1407	28.1	0.56 (0.46‐0.68)	0.46 (0.38‐0.57)
Primary CVD prevention	91	12.5	830	16.6	0.59 (0.46‐0.75)	0.51 (0.40‐0.65)
Secondary CVD prevention	55	7.6	577	11.5	0.52 (0.38‐0.69)	0.41 (0.30‐0.55)
Remaining current users^c^	66	9.1	730	14.6	0.46 (0.35‐0.61)	0.31 (0.23‐0.42)
Recent use	26	3.6	146	2.9	0.93 (0.60‐1.43)	0.66 (0.42‐1.02)
Past use	47	6.5	313	6.3	0.80 (0.58‐1.10)	0.57 (0.41‐0.79)
Dose^d^						
75 mg	143	19.7	1377	27.5	0.56 (0.46‐0.69)	0.46 (0.38‐0.57)
150 mg	2	0.3	14	0.3	0.77 (0.17‐3.39)	0.78 (0.17‐3.62)
300 mg	1	0.1	16	0.3	0.34 (0.04‐2.55)	0.28 (0.04‐2.15)
Duration of low‐dose aspirin use^d^						
>1 to <3 years	56	7.7	693	13.9	0.42 (0.31‐0.56)	0.34 (0.25‐0.46)
>3 to <5 years	63	8.7	508	10.2	0.68 (0.51‐0.90)	0.57 (0.43‐0.77)
≥5 years	27	3.7	206	4.1	0.76 (0.50‐1.16)	0.66 (0.43‐1.01)
Concomitant use of low‐dose aspirin and another medication^d^ ^,^ ^e^						
Low‐dose aspirin plus clopidogrel	7	1.0	43	0.9	0.90 (0.40‐2.02)	0.62 (0.27‐1.42)
Low‐dose aspirin plus NSAID	7	1.0	95	1.9	0.39 (0.18‐0.85)	0.27 (0.12‐0.58)
Low‐dose aspirin plus PPI	97	13.3	454	9.0	2.77 (2.09‐3.67)	1.94 (1.45‐2.60)

Abbreviations: CI, confidence interval; CVD, cardiovascular disease; GP, general practitioner; OR, odds ratio; PPI, proton pump inhibitor; NSAID, nonsteroidal anti‐inflammatory drug.

a Adjusted by the matching factors: sex, age and calendar year.

b Adjusted by the matching factors: sex, age, and calendar year, and by smoking and number of GP visits in the previous year.

c Current users of low‐dose aspirin with less than 1 year duration of use.

d Among current users of low‐dose aspirin with ≥1 year duration of use.

e Compared to no use of either drug in the year before the index date.

### 3.3 Low‐dose aspirin use and risk of oesophageal cancer

Associations between low‐dose aspirin and risk of oesophageal cancer are shown in Table 4. Compared to nonuse of low‐dose aspirin, current use of low‐dose aspirin was associated with a 41% reduced risk of oesophageal cancer (OR 0.59, 95% CI: 0.51‐0.69). Findings relating to dose, duration and primary/secondary CVD prevention were similar to those seen for gastric cancer, including in the lag‐time analyses (Table S4), with little change seen in the OR from the main (current use) estimate. Also, as seen with gastric cancer, a reduced risk of oesophageal cancer was seen when low‐dose aspirin was used concomitantly with clopidogrel (OR 0.23, 95% CI: 0.10‐0.56 vs nonuse of both drugs) or with NSAIDs (OR 0.58, 95% CI: 0.36‐0.96 vs nonuse of both drugs), and an increased risk of oesophageal cancer was seen with concomitant of a PPI (OR 3.18, 95% CI: 2.55‐3.95).

**TABLE 4:** Odds ratio (95% CIs) for the association between low‐dose aspirin and the risk of oesophageal cancer

	Cases (n = 1394)		Controls (n = 5000)			
Low‐dose aspirin	n	%	n	%	OR (95% CI)^a^	OR (95% CI)^b^
Recency of use						
Nonuse	829	59.5	2555	51.1	1.0 (reference)	1.0 (reference)
Current use	331	23.7	1397	27.9	0.73 (0.63‐0.84)	0.59 (0.51‐0.69)
Primary CVD prevention	180	12.9	821	16.4	0.67 (0.56‐0.80)	0.56 (0.46‐0.67)
Secondary CVD prevention	151	10.8	576	11.5	0.80 (0.66‐0.98)	0.64 (0.52‐0.78)
Remaining current users^c^	103	7.4	625	12.5	0.49 (0.39‐0.61)	0.33 (0.26‐0.42)
Recent use	63	4.5	133	2.7	1.43 (1.04‐1.95)	0.95 (0.69‐1.31)
Past use	68	4.9	290	5.8	0.71 (0.54‐0.94)	0.49 (0.37‐0.65)
Dose^d^						
75 mg	303	21.7	1323	26.5	0.70 (0.60‐0.81)	0.57 (0.49‐0.67)
150 mg	3	0.2	14	0.3	0.65 (0.19‐2.26)	0.58 (0.16‐2.09)
300 mg	8	0.6	16	0.3	1.52 (0.65‐3.56)	1.02 (0.42‐2.44)
Duration of low‐dose aspirin use^d^						
>1 to <3 years	145	10.4	680	13.6	0.64 (0.52‐0.78)	0.51 (0.41‐0.62)
>3 to <5 years	95	6.8	372	7.4	0.79 (0.62‐1.00)	0.64 (0.55‐0.92)
≥5 years	91	6.5	345	6.9	0.83 (0.65‐1.07)	0.71 (0.55‐0.92)
Concomitant use of low‐dose aspirin and another medication^d^ ^,^ ^e^						
Low‐dose aspirin plus clopidogrel	6	0.4	54	1.1	0.34 (0.15‐0.80)	0.23 (0.10‐0.56)
Low‐dose aspirin plus NSAID	21	1.5	84	1.7	0.74 (0.45‐1.20)	0.58 (0.36‐0.96)
Low‐dose aspirin plus PPI	234	16.8	426	8.5	4.21 (3.41‐5.19)	3.18 (2.55‐3.95)

Abbreviations: CI, confidence interval; CVD, cardiovascular disease; GP, general practitioner; OR, odds ratio; PPI, proton pump inhibitor; NSAID, nonsteroidal anti‐inflammatory drug.

a Adjusted by the matching factors: sex, age and calendar year.

b Adjusted by the matching factors: sex, age, and calendar year, and by smoking and number of GP visits in the previous year.

c Current users of low‐dose aspirin with less than 1 year duration of use.

d Among current users of low‐dose aspirin with ≥1 year duration of use.

e Compared to no use of either drug in the year before the index date.

## 4 DISCUSSION

Our large population‐based observational study found that use of low‐dose aspirin for at least 1 year was associated with a significant 54% reduction in the risk of gastric cancer and a 41% reduced risk of oesophageal cancer when compared to nonuse of low‐dose aspirin, consistent with the substantial reductions in risk of these two cancers seen in previous studies.^3^, ^20^, ^21^, ^22^, ^23^, ^24^, ^25^ The reductions in risk were seen at the lowest dose of aspirin evaluated (75 mg), and effect sizes were broadly consistent with increasing lengths of low‐dose aspirin use with no clear change seen between durations of 1‐3, 3‐5 or >5 years.

This beneficial effect of low‐dose aspirin after a few years of therapy as observed in our study can be explained by an inhibitory effect of low‐dose aspirin on the development of existing tumours and/or metastasis. This is supported by both meta‐analysis of CVD RCTs and observational studies for CRC,^2^, ^4^ and is the reason postulated for at least part of the early reduction of cancer deaths in the trials. Furthermore, Rothwell et al reported that allocation to low‐dose aspirin improved overall survival in patients who developed adenoma during the trials.^2^ An effect of low‐dose aspirin later in the adenoma‐carcinoma sequence is also supported by experimental data showing the central role of platelets in this context,^33^, ^34^ in addition to animal studies showing the significant inhibition of tumour cell development, metastasis and angiogenesis after pharmacological inhibition of thromboxane synthase.^35^ In Rothwell's meta‐analysis of three aspirin CVD trials, the statistical evidence of an effect of low‐dose aspirin in reducing gastric and oesophageal cancer risk was strongest after 10 to 20 years follow‐up. The shorter follow‐up duration in our study limited observation of any latent effects indicative of an effect of low‐dose aspirin early in the adenoma‐carcinogenesis sequence. However, the current body of evidence suggests that the chemopreventive action of low‐dose aspirin on gastrointestinal cancer acts both early and late in the adenoma‐carcinogenesis pathway.^8^ It should also be kept in mind that RCT data are limited by the assessment of low‐dose aspirin at the start of the trial period meaning that exposure could have changed substantially (eg, stopped among members of the low‐dose aspirin arm and/or initiated among members of the placebo arm) over the long post‐trial follow‐up periods.

Recent meta‐analyses, including both RCT and observational data, have suggested a possible duration of use effect of low‐dose aspirin on gastric cancer risk. Ye et al^21^ reported that significant reductions in risk were only seen after at least 5 years of use, while Huang et al^23^ observed a 5% risk reduction with every 2‐year increment in duration. Yet, a large cohort study in Hong Kong published last year reported no duration of use effects,^24^ and data on duration effects for oesophageal cancer are lacking. Our finding that a dose of aspirin of 75 mg/day was sufficient to observe the substantial reductions in gastric/oesophageal cancer risk is in line with the low doses shown to be beneficial in reducing the risk of gastrointestinal cancers in clinical trials and previous observational studies.^4^, ^5^, ^8^ We observed the substantial reduction in gastric/oesophageal cancer risk in both primary and secondary CVD prevention populations, as we have also shown previously for CRC.^4^, ^5^ Whether increasing evidence in support of low‐dose aspirin for chemoprophylaxis of gastric and oesophageal cancer in addition to CRC will further improve the overall balance of benefits and harms toward favouring low‐dose aspirin in primary CVD prevention is a question that will be answered in time. Expert opinion and clinical guidelines on this topic have been evolving in recent years as new robust data on cancer, CVD and bleeding outcomes emerge.^36^, ^37^, ^38^, ^39^, ^40^ Incorporating data on gastric/oesophageal cancer prophylaxis into benefit‐risk assessments of low‐dose aspirin for both CVD and cancer prevention would require greater consensus around any duration of effects. Data applicable to patient subgroups would also be beneficial. Interestingly, the ASCEND RCT of 100 mg daily aspirin vs placebo among individuals with diabetes but no evident CVD found no significant difference in the incidence of gastrointestinal cancers as a group after 7.4 years’ follow‐up. However, the trial only had 60% power to detect the 30% hypothesised difference between study arms,^41^ and results from longer‐term follow‐up are awaited.

Strengths of our study include the large sample representative of the population from which it was drawn, meaning our results are generalizable to the UK population. The study population reflected users and nonusers of low‐dose aspirin in clinical practice including the elderly and those with multiple comorbidities. Our matched cohort design helped to minimise bias from differences between low‐dose aspirin users and nonusers at the start date. Nested case‐control analyses enabled low‐dose aspirin exposure to be accurately assessed, and lag‐time analyses found our findings to be robust. We explored many potential confounders with adjustment made for variables found to be confounders in our analyses, yet we acknowledge that residual confounding cannot be completely ruled out, for example, from unknown confounders. The stepwise process for case confirmation involved manual review of patients’ EHRs, and although we did not validate our cases of gastric/oesophageal cancer through linkage to hospital data, in a previous study for CRC restricted to individuals also in the Hospital Episode Statistics (HES) database, the false negative rate for CRC (using HES as gold standard) was only 6.1%.^4^ Hence, it is likely that THIN is also a valid data source to identify cases of gastric/oesophageal cancer. Linkage to UK cancer registry data was not possible because this is not currently available for THIN. We were unable to evaluate associations according to histologic type of cancer or stage because this information is not systematically recorded in THIN. Although an increased risk of both gastric and oesophageal cancer was seen among low‐dose aspirin users with concomitant PPI use, this finding should be interpreted very cautiously because of the likelihood of protopathic bias. Proton pump inhibitors are commonly prescribed for acid‐reflux or similar symptoms, which are also early indicators of these two gastrointestinal cancers.

Our findings add to the existing evidence that low‐dose aspirin is associated with a substantial reduction in the risk of gastric/oesophageal cancer in the general population. Further population‐based research is needed to clarify any associations with duration of use. Future research into the effects of low‐dose aspirin on gastric and oesophageal cancer risk by stage at diagnosis, as well as potential effects on other cancers, would also be beneficial lines of investigation. This would further help our understanding of the mechanism(s) of aspirin‐mediated chemoprotection in the gastrointestinal tract and other locations.

## CONFLICT OF INTEREST

LAGR works for CEIFE, which has received research funding from Bayer AG. LAGR has also received honoraria for serving on advisory boards for Bayer AG. MS‐G and PV are employees of Bayer AG. LCS has no conflicts of interest.

## ETHICS STATEMENT

Acquisition of THIN data was provided to the Centro Español de Investigación Farmacoepidemiológica (CEIFE) by IQVIA under the UK's National Health Service South‐East multicenter research committee approval in 2003. The study protocol was approved by an Independent Scientific Research Committee for THIN (reference 17THIN059_A1). No individual patient consent was required because the study used de‐identified data provided by patients as a part of their routine primary care.

## Supporting information


**Appendix S1** **:** Supporting information

## Data Availability

Data are available from the corresponding author upon reasonable request.
