# Supplementary material for: Low‐dose aspirin and risk of gastric and oesophageal cancer: A population‐based study in the United Kingdom using The Health Improvement Network
Source: Int J Cancer. 2020 May 7;147(9):2394–404. doi: 10.1002/ijc.33022 (PMC7540378; doi:10.1002/ijc.33022)
Supplement: Supplementary file 1 — Appendix S1 : Supporting information [file IJC-147-2394-s001.pdf]

SUPPLEMENT

Low-dose Aspirin and Risk of Gastric and Oesophageal Cancer: A Population-Based Study in the United Kingdom using The Health Improvement Network

Luis A García Rodríguez , Montse Soriano-Gabarró, Pareen Vora, Lucía Cea Soriano

TABLE OF CONTENTS

- **Supplementary Methods:** Identification of patients censored by having a record of another cancer
- **Supplementary Table 1**. Read codes for gastric cancer.
- **Supplementary Table 1**. Read codes for oesophageal cancer.
- **Supplementary Table 3**. ORs (95% CIs) for the association between low-dose aspirin and gastric cancer using the 1-year backdated index date.
- **Supplementary Table 4**. ORs (95% CIs) for the association between low-dose aspirin and oesophageal cancer using the 1-year backdated index date.
- **Supplementary Figure:** Flowchart depicting the study design

Supplementary Methods: Identification of patients censored by having a record of another cancer

To identify cases with a record of another primary cancer before the diagnosis or gastric/oesophageal cancer, we searched patients' EHRs for Read codes suggestive of cancer, e.g. codes for primary/secondary/unspecific malignancy, during the follow-up period but before the index date and manually reviewed their records to categorise them as confirmed or non-confirmed cases through the following stepwise process.

- Firstly, if the previous record suggestive of another cancer was for basal cell carcinoma, 'adenocarcinoma not otherwise specified' or was unspecific, e.g. seen in oncology clinic, carcinoma with unspecified site, cancer monitoring, then the patient was confirmed as a case of gastric/oesophageal cancer.
- Secondly, if the previous record was within 30 days before the record entry of gastric/oesophageal cancer, then we manually reviewed their EHR to decide case status (confirmed or non-confirmed).
- Lastly, all remaining patients with a previous record suggestive of another cancer before the record of gastric/oesophageal cancer were excluded.

Supplementary Table 1. Read codes for gastric cancer.

Read	Description
B11..00	Malignant neoplasm of stomach
B11..11	Gastric neoplasm
B110.00	Malignant neoplasm of cardia of stomach
B110000	Malignant neoplasm of cardiac orifice of stomach
B110100	Malignant neoplasm of cardio-oesophageal junction of stomach
B110111	Malignant neoplasm of gastro-oesophageal junction
B110z00	Malignant neoplasm of cardia of stomach NOS
B111.00	Malignant neoplasm of pylorus of stomach
B111000	Malignant neoplasm of prepylorus of stomach
B111100	Malignant neoplasm of pyloric canal of stomach
B111z00	Malignant neoplasm of pylorus of stomach NOS
B112.00	Malignant neoplasm of pyloric antrum of stomach
B113.00	Malignant neoplasm of fundus of stomach
B114.00	Malignant neoplasm of body of stomach
B115.00	Malignant neoplasm of lesser curve of stomach unspecified
B116.00	Malignant neoplasm of greater curve of stomach unspecified
B117.00	Malignant neoplasm, overlapping lesion of stomach
B11y.00	Malignant neoplasm of other specified site of stomach
B11y000	Malignant neoplasm of anterior wall of stomach NEC
B11y100	Malignant neoplasm of posterior wall of stomach NEC
B11yz00	Malignant neoplasm of other specified site of stomach NOS
B11z.00	Malignant neoplasm of stomach NOS
B118.00	Siewert type II adenocarcinoma
B119.00	Siewert type III adenocarcinoma
B902000	Neoplasm of uncertain behaviour of stomach

Supplementary Table 2. Read codes for oesophageal cancer.

Read	Description
B10..00	Malignant neoplasm of oesophagus
B100.00	Malignant neoplasm of cervical oesophagus
B101.00	Malignant neoplasm of thoracic oesophagus
B102.00	Malignant neoplasm of abdominal oesophagus
B103.00	Malignant neoplasm of upper third of oesophagus
B104.00	Malignant neoplasm of middle third of oesophagus
B105.00	Malignant neoplasm of lower third of oesophagus
B106.00	Malignant neoplasm, overlapping lesion of oesophagus
B107.00	Siewert type I adenocarcinoma
B10y.00	Malignant neoplasm of other specified part of oesophagus
B10z.00	Malignant neoplasm of oesophagus NOS
B10z.11	Oesophageal cancer

Supplementary Table 3. ORs (95% CIs) for the association between low-dose aspirin and gastric cancer using the 1 year backdated index date.

	Controls N=5000		Cases N=727		OR (95% CI)[*]	OR (95% CI)[†]
	n	%	n	%		
**Low-dose aspirin**						
Non-use	2212	44.2	414	56.9	1.0 (reference)	1.0 (reference)
Current use	1639	32.8	186	25.6	0.60 (0.50–0.72)	0.47 (0.39–0.57)
Duration ≤1 year	502	10.0	38	5.2	0.38 (0.27–0.54)	0.28 (0.20–0.40)
Duration ≥1 year	1137	22.7	148	20.4	0.70 (0.57-0.86)	0.57 (0.46-0.70)
Duration >1 to <3 years	575	11.5	71	9.8	0.65 (0.49–0.85)	0.51 (0.39–0.67)
Duration ≥3 years	562	11.2	77	10.6	0.76 (0.58–1.00)	0.64 (0.49–0.84)
Recent use	128	2.6	15	2.1	0.61 (0.35–1.06)	0.49 (0.28–0.86)
Past use	236	4.7	34	4.7	0.77 (0.53–1.11)	0.61 (0.42–0.90)
Remaining category[‡]	785	15.7	78	10.7	0.49 (0.38–0.64)	0.33 (0.25–0.43)

[*]Adjusted by the matching factors: sex, age and calendar year

[‡]Adjusted by the matching factors: sex, age and calendar year, and also the number of GP visits in the year before the index date, smoking status and low-dose aspirin.

[‡]Remaining: individuals whose first prescription for low-dose aspirin was in the year before the original non-backdated index date.

CI, confidence interval; GP, general practitioner; OR, odds ratio

Supplementary Table 4. ORs (95% CIs) for the association between low-dose aspirin and oesophageal cancer using the 1 year backdated index date.

	Controls N=5000		Cases N=1394		OR (95% CI)*	OR (95% CI)†
	n	%	n	%		
**Low-dose aspirin**						
Non-use	2373	47.5	778	55.8	1.0 (reference)	1.0 (reference)
Current use	1641	32.8	398	28.6	0.73 (0.64–0.84)	0.58 (0.50–0.67)
Duration ≤1 year	518	10.4	99	7.1	0.56 (0.44–0.71)	0.43 (0.34–0.54)
Duration ≥1 year	1,123	22.5	299	21.5	0.82 (0.70–0.95)	0.65 (0.56–0.77)
Duration >1 to <3 years	550	11.0	135	9.7	0.74 (0.60–0.91)	0.57 (0.46–0.71)
Duration ≥3 years	573	11.5	164	11.8	0.90 (0.74–1.09)	0.74 (0.60–0.90)
Recent use	106	2.1	27	1.9	0.77 (0.50–1.19)	0.60 (0.39–0.94)
Past use	217	4.3	64	4.6	0.89 (0.67–1.20)	0.69 (0.51–0.93)
Remaining category‡	663	13.3	127	9.1	0.56 (0.45–0.69)	0.37 (0.30–0.47)

*Adjusted by the matching factors: sex, age and calendar year

‡Adjusted by the matching factors: sex, age and calendar year, and also the number of GP visits in the year before the index date, smoking status and low-dose aspirin.

‡Remaining: individuals whose first prescription for low-dose aspirin was in the year before the original non-backdated index date.

CI, confidence interval; GP, general practitioner; OR, odds ratio

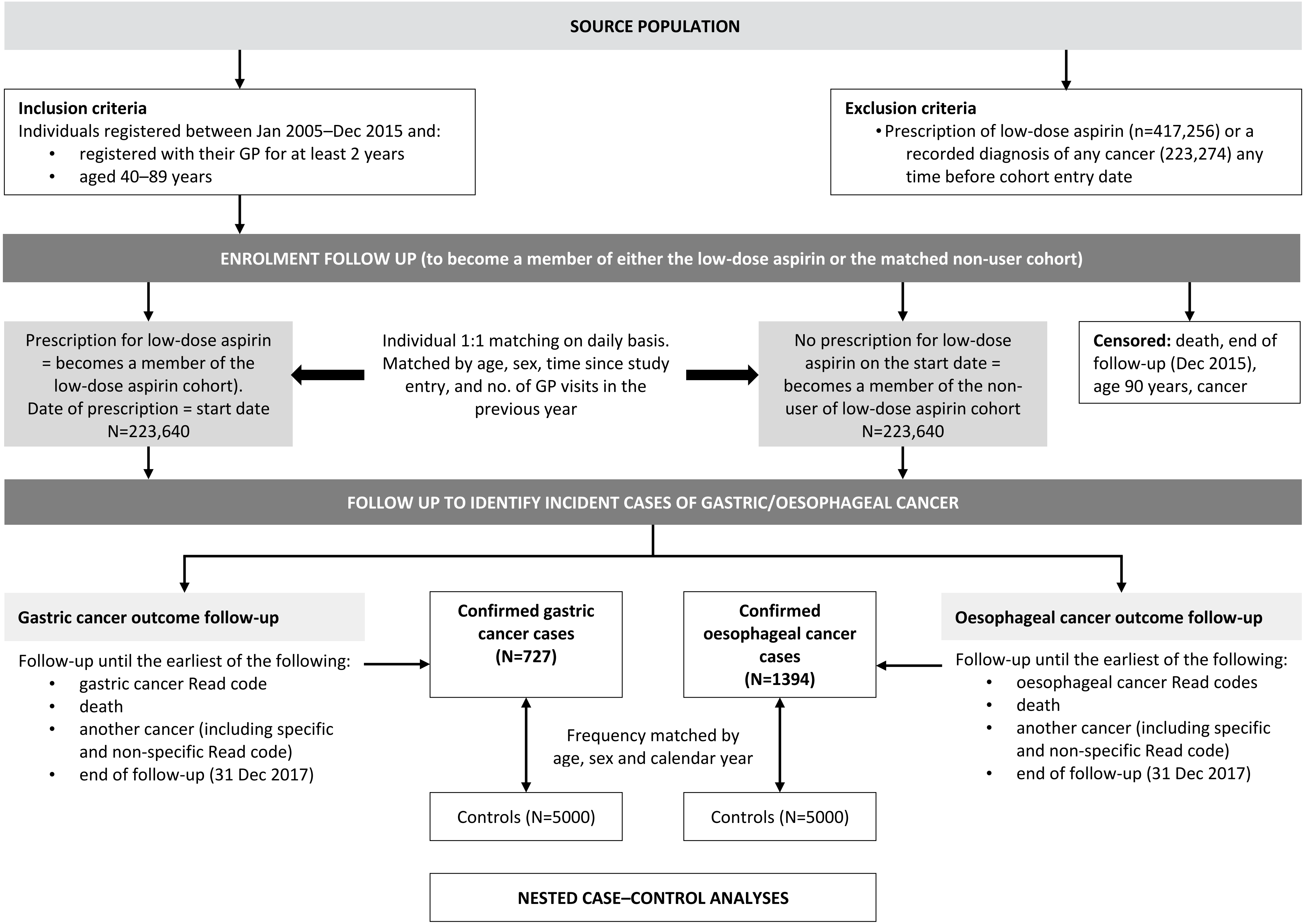

Supplementary Figure: Flowchart depicting the study design.
